# XPS, TDS, and AFM studies of surface chemistry and morphology of Ag-covered L-CVD SnO_2_ nanolayers

**DOI:** 10.1186/1556-276X-9-260

**Published:** 2014-05-24

**Authors:** Monika Kwoka, Luca Ottaviano, Piotr Koscielniak, Jacek Szuber

**Affiliations:** 1 Institute of Electronics, Silesian University of Technology, Gliwice 44-100, Poland; 2 CNR- SPIN & Department of Physics and Chemical Sciences University of L’Aquila, L’Aquila 67100, Italy

**Keywords:** Tin dioxide SnO_2_, L-CVD thin films, Ag-doping, Surface chemistry, Surface morphology, XPS, TDS, AFM

## Abstract

This is well known that the selectivity and sensitivity of tin dioxide (SnO_2_) thin film sensors for the detection of low concentration of volatile sulfides such as H_2_S in air can be improved by small amount of Ag additives. In this paper we present the results of comparative X-ray photoelectron spectroscopy (XPS), thermal desorption spectroscopy (TDS), and atomic force microscopy (AFM) studies of the surface chemistry and morphology of SnO_2_ nanolayers obtained by laser-enhanced chemical vapor deposition (L-CVD) additionally covered with 1 monolayer (ML) of Ag. For as deposited SnO_2_ nanolayers, a mixture of tin oxide (SnO) and tin dioxide (SnO_2_) with the [C]/[Sn] ratio of approximately 1.3 was observed. After dry air exposure, the [O]/[Sn] ratio slightly increased to approximately 1.55. Moreover, an evident increasing of C contamination was observed with [C]/[Sn] ratio of approximately 3.5. After TDS experiment, the [O]/[Sn] ratio goes back to 1.3, whereas C contamination evidently decreases (by factor of 3). Simultaneously, the Ag concentration after air exposure and TDS experiment subsequently decreased (finally by factor of approximately 2), which was caused by the diffusion of Ag atoms into the subsurface layers related to the grain-type surface morphology of Ag-covered L-CVD SnO_2_ nanolayers, as confirmed by XPS ion depth profiling studies. The variation of surface chemistry of the Ag-covered L-CVD SnO_2_ after air exposure observed by XPS was in a good correlation with the desorption of residual gases from these nanolayers observed in TDS experiments.

## Background

Tin dioxide (SnO_2_) has drawn a great interest, among other oxides, related the response to oxidizing and reducing gases [1]. Nowadays the research is focusing on nanostructured materials, among other nanowires, because they have a large surface-to-volume ratio and show enhanced chemical stability and electrical performances [2,3]. However, thin film technology is a core high-yield fabrication method for real-world sensors because of the main advantages such as low power consumption.

In order to improve selectivity and sensitivity of the SnO_2_ thin films-based gas sensors, various dopants are used. It is well known that SnO_2_ thin film sensors doped with Ag additives are very sensitive to low concentration of volatile sulfides such as H_2_S in air [4]. Up to now, this mechanism is not fully clear. However, it is certain that the influence of dopants like Ag must be related to the variation of the surface chemistry, morphology, and electronic properties of SnO_2_ thin films.

Apart from the above, one of the most technologically relevant and still scarcely addressed problem in the world of real sensors is their degradation in time. This is why the aging effect of SnO_2_ thin films after their air exposure related mainly to the undesired and uncontrolled C carbon contamination coming from CO_2_ in the atmosphere is also of great importance [5]. This is even more serious when SnO_2_ nanostructures are covered with Ag additives.

The aging problem in the case of pure SnO_2_ nanolayers prepared by laser-enhanced chemical vapor deposition (L-CVD) method has been already addressed in our recent studies [5,6]. The main observation from this study was that long-term exposure (aging) in dry air of L-CVD SnO_2_ thin films caused them to be covered with a large amount of undesired carbon species. They can be reduced after their ultrahigh vacuum (UHV) annealing up to 670 K. However, X-ray photoelectron spectroscopy (XPS) method cannot give any information concerning the forms of desorbing species. One can expect that this desorption process can be affected by the presence of Ag surface additives. This type of information can be obtained using, for instance, thermal desorption spectroscopy (TDS) method. This is why in this paper, we present the results of a comparative study of the surface chemistry and morphology of Ag-covered L-CVD SnO_2_ nanolayers carried out by XPS in combination with TDS, respectively. Such combination of methods allowed us to control simultaneously the variation of surface chemistry (including stoichiometry and purity) of Ag-covered L-CVD SnO_2_ nanolayers by XPS, during the desired desorption of surface atoms (including contaminations) from these nanolayers. A special emphasis was given to the analysis of behavior of C contamination from the air interacting with their surface. Moreover, for the additional control of surface morphology of Ag-covered L-CVD SnO_2_ nanolayers, the atomic force microscopy (AFM) method was applied.

## Methods

Ag-covered L-CVD SnO_2_ nanolayers were deposited at ENEA (Ente Nazionale Energie Alternative) Centre, Frascati, Italy, on Si(100) substrates at room temperature, which were firstly cleaned by UHV (10^−7^ Pa) annealing at 940°C. During the deposition tetramethyltin (TMT)-O_2_ mixture with flows of 0.2 and 5 sccm, respectively, was used and irradiated with pulsed laser beam (5 Hz, 20 mJ/cm^2^ flux density) of ArF excimer (193 nm) laser (Lambda Physik, LPX 100 model; Göttingen, Germany) set in a perpendicular geometry. The thickness of SnO_2_ nanolayers was 20 nm after 60 min of deposition, as determined *in situ*, with a quartz crystal microbalance (QMB). Subsequently, 1 ML Ag ultrathin film was deposited by thermal evaporation in UHV on the freshly deposited (as-prepared) SnO_2_ nanolayers. The freshly deposited samples were then *in situ* characterized by X-ray photoelectron spectroscopy (XPS) using a PHI model spectrometer equipped with X-ray lamp (Al Kα 1486.6 eV) and double-pass cylindrical mirror analyzer (DPCMA) model 255G.

The surface chemistry including contaminations of the abovementioned Ag-covered SnO_2_ nanolayers after dry air exposure was controlled sequentially by XPS. In order to detect the surface active gas species adsorbed at the surface of Ag-covered L-CVD SnO_2_ nanolayers after air exposure, a subsequent thermal desorption experiment was performed in line with a mass spectrometry (MS) to measure the desorbed products.

To check the aging effects, the XPS experiments were carried out with a SPECS model XPS spectrometer (SPECS Surface Nano Analysis GmbH, Berlin, Germany) equipped with the X-ray lamp (Al K_α_ 1,486.6 eV; XR-50 model) and a concentric hemispherical analyzer (PHOIBOS-100 model). The system was operating at 10^−7^ Pa. XPS ion depth profiling experiments were performed using a differentially pumped ion gun (IQE-12/38 model) working at 3 keV. All the reported binding energies (BE) data have been calibrated to the Au4*f* peak at 84.5 eV.

The TDS measurements were performed in the sample preparation chamber equipped with a residual gas analyzer (Stanford RGA100 model; Stanford Research Systems, Sunnyvale, CA, USA) combined with a temperature programmable control unit-dual-regulated power supply (OmniVac PS REG120, Kaiserslautern, Germany). During the thermal desorption studies, the temperature increased by 6°C per minute in the range of 50°C to 350°C to avoid undesired decomposition of L-CVD SnO_2_ nanolayers, and the TDS spectra of H_2_, H_2_O, O_2_, and CO_2_ have been acquired and then corrected by the corresponding gas ionization probability.

The AFM experiments were performed at the CASTI Centre, L’Aquila University, Coppito, Italy, using the AFM Digital Dimension 5000 with NANOSCOPE IV controller from Digital Instruments (Veeco Metrology Group; Santa Barbara, CA, USA), operating in tapping mode. In these AFM measurements, the sharpened silicon probes of nominal tip radius of curvature 20 to 30 nm were used for imaging. A silicon tip is scanned across the surface of a sample at a constant force of 16 N/m. The operating head scans the substrate up to 90 μm in X-Y and up to 6 μm in Z. This scanner includes a piezoelectric tube scanner, a laser, and a quadrate optical detector. Set points were chosen close to the free oscillation amplitude to minimize forces exerted on the interfacial species. Effective resonance frequencies inside the fluid were approximately 300 kHz. The maximum spatial resolution (1 nm) and vertical resolution (0.1 A) allows the revealing of the surface structure at atomic level. The AFM image analysis was carried out using commercial WSxM 4.0 (Nanotec Electronica, Madrid, Spain) software procedures to determine surface roughness that is represented by root mean square (RMS) parameter and the values of average and maximum grain height. Other experimental details have been described in [7,8].

## Results and discussion

**Figure 1:**
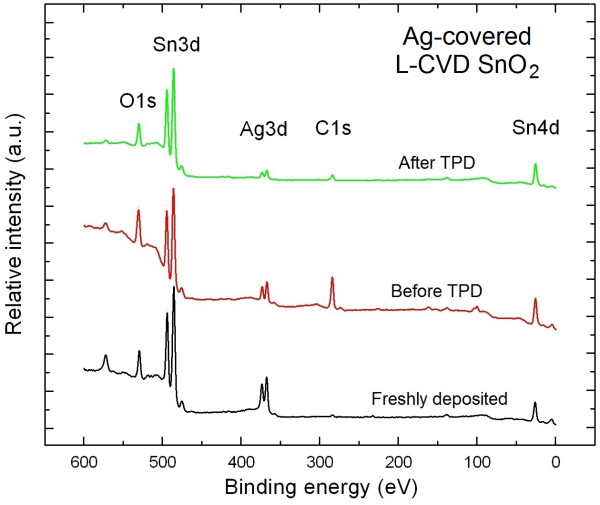
**XPS survey spectra of Ag-covered L-CVD SnO**_**2 **_**nanolayers and subsequent processes.**

Figure 1 shows the XPS survey spectra of the Ag-covered L-CVD SnO_2_ nanolayers after the technological procedure described in Section ‘Methods’.

With decreasing binding energy, the following core levels are verified: O1*s*, Sn3*d* doublet, Ag3*d* doublet, C1*s*, and Sn4*d*. It was the base for determination of their surface chemistry (including stoichiometry and contaminations) based on the atomic sensitivity factor (ASF) approach [9] using the recently described procedure [5,6].

The Ag-covered L-CVD SnO_2_ nanolayers freshly deposited on atomically clean Si(100) substrate were treated as a reference sample in our studies. They exhibit good purity because (apart from a very weak C1*s* peak at signal-to-noise (S/N) ratio of approximately 2) only the O1*s*, Sn3*d*, Ag3*d* related core level XPS peaks were measured. The shoulders at the low binding energy (BE) of Ag and Sn core level doublets are satellite features owed to the use of the non-monochromatized X-ray radiation. For this freshly deposited Ag-covered L-CVD SnO_2_ nanolayers, the relative [O]/[Sn] concentration was equal to 1.30 ± 0.05. This means that these nanolayers are a mixture of SnO and SnO_2_ in about 2:1 ratio with dominance of SnO in the layer. Using the same analytical procedure, the relative [Ag]/[Sn] concentration was determined as equal to 0.50 ± 0.05. It corresponds to about 0.5 nm (1 ML) of Ag atoms deposited at the top, as estimated also by the QMB. More in general the results of quantitative elemental surface of the spectra of Figure 1 are reported in Table 1.

**Table 1:** **Elemental relative concentration of Ag-covered L-CVD SnO**_**2 **_**nanolayers at different steps of the procedure**

**Ag-doped L-CVD SnO**_**2 **_**nanolayer procedures**	**Relative concentration**		
	**[O]/[Sn]**	**[C]/[Sn]**	**[Ag]/[Sn]**
Freshly deposited	1.30 ± 0.05	0.30 ± 0.05	0.50 ± 0.05
After exposure in dry air	1.55 ± 0.05	3.50 ± 0.05	0.25 ± 0.05
After subsequent TDS	1.30 ± 0.05	1.10 ± 0.05	0.15 ± 0.05

**Figure 2:**
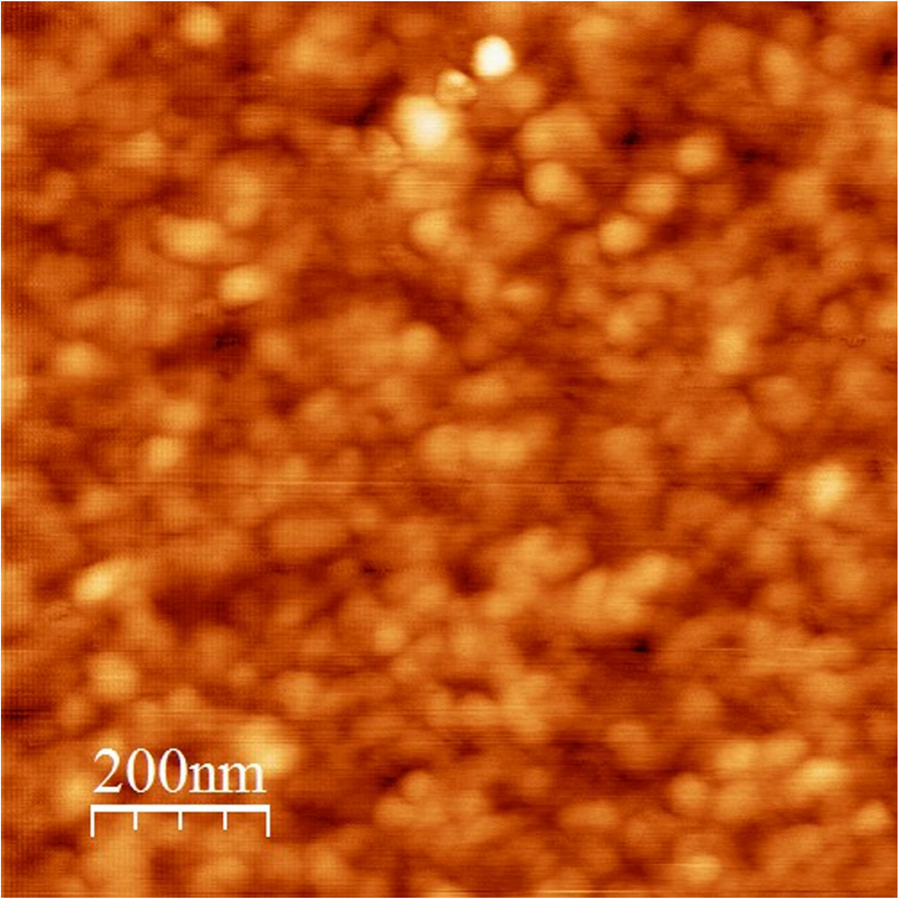
**AFM image of the Ag-covered L-CVD SnO**_**2 **_**nanolayers.**

At the next step of our studies, the freshly deposited Ag-covered L-CVD SnO_2_ nanolayers were long-term exposed (aged) in dry air atmosphere at room temperature and this caused evident changes in their surface chemistry. Firstly, the relative [O]/[Sn] concentration reached the value of 1.55 ± 0.05. Likely, the increased O concentration after air exposure is due to the surface contaminations containing oxygen (CO_2_, H_2_O), what will be discussed and analyzed later on the basis of TDS spectra. Simultaneously, the relative [Ag]/[Sn] concentration evidently (more than twice) decreased reaching value 0.25 ± 0.05. At this point, we presume that to some extent, the even distribution of Ag atoms at the surface/subsurface of SnO/SnO_2_ films in the form of very flat 3D (2D) nanoparticles/clusters is related to the aging effect. However, what is most important to notice is that after this procedure, remarkable C contamination was detected, observed in the form of a strong C1*s* XPS peak shown in the survey spectra in Figure 1. The corresponding relative [C]/[Sn] concentration was equal to 3.50 ± 0.05. This value is one order larger than for the freshly deposited Ag-covered L-CVD SnO_2_ nanolayers. However, it should be pointed out at this moment that this high C contamination observed by XPS method concerns only the very thin near-surface region of the investigated films because the information depth for SnO_2_ is about 4 nm. Moreover, our recent depth profiling XPS experiments showed that C contamination is mostly located only at the topmost 2 to 3 atomic layers because going down in depth, the relative concentration of [C]/[Sn] was about 0.1, which was almost constant up to the Si substrate. This is strongly related to the grain-type surface morphology of Ag-covered L-CVD SnO_2_ nanolayers with the grains standing up in respect to the surface plane, as observed in the AFM image shown in Figure 2.

Very precise standard AFM depth profiling analysis (with DI software) showed that the maximum grain height and the maximum grain width for these nanolayers were estimated as equal to about 3 and 30 nm, respectively. In turn their average roughness was about 0.5 nm, which was very similar to the pure L-CVD SnO_2_ nanolayers, as determined in our recent AFM studies [8]. It means that deposition of 1 ML of Ag does not significantly modify the surface/subsurface morphology of L-CVD SnO_2_ nanolayers.

**Figure 3:**
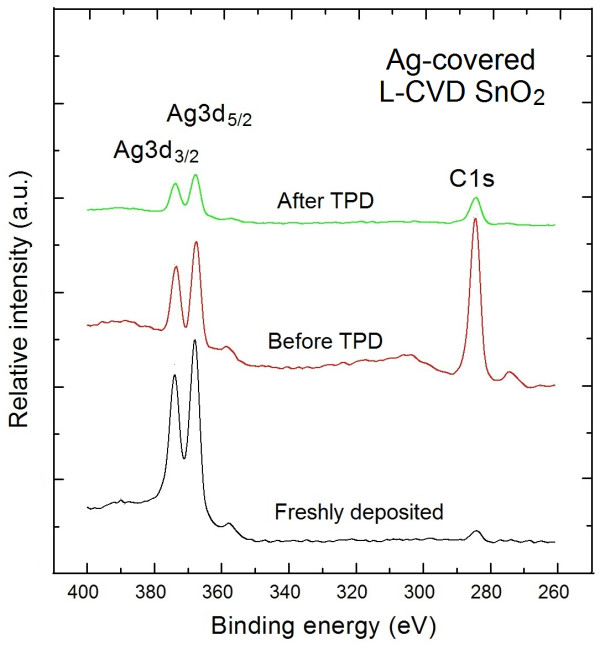
**XPS Ag3 *d *-C1** ***s *** **spectral windows.**

The surface chemistry, including stoichiometry and contaminations, of the Ag-covered L-CVD SnO_2_ nanolayers after long-term exposure (aging) in dry air atmosphere was dramatically changed after registration of TDS spectra, as shown in respective XPS survey spectrum in Figure 3.

Firstly, the relative [O]/[Sn] concentration evidently decreased reaching a value of 1.30 ± 0.05. This is probably related to the fact that the contaminations at the surface of Ag-covered L-CVD SnO_2_ nanolayers after air exposure containing oxygen (CO_2_, H_2_O) physically bounded to their surface are removed during the TDS experiment. This is also related to the evident decreasing of the C contamination because the corresponding [C]/[Sn] ratio reached a value of 1.10 ± 0.05. This value is more than twice smaller than for the pure L-CVD SnO_2_ thin films after similar long-term aging [7] and subsequent UHV annealing. It indicates that this procedure is even more useful for remarkable decreasing of surface C contaminations for the Ag-covered L-CVD SnO_2_ nanolayers after long-term aging in dry air atmosphere with respect to the pure L-CVD SnO_2_ nanolayers. A similar effect was observed by Maffeis et al. [10] for nanocrystalline SnO_2_ gas sensor layers. This drastic decreasing of C contamination at the top of Ag-covered L-CVD SnO_2_ nanolayers after TDS experiment is related to the fact that the 3D/2D Ag nanoparticles/clusters are distributed within the subsurface layers of Ag-covered L-CVD SnO_2_ nanolayers because they exhibit a natural tendency to diffuse into the nanolayer up to the Si substrate, which was independently confirmed by XPS depth profiling analysis in our recent studies [11]. What is also important, Ag islands (nanoclusters) at the top of L-CVD SnO_2_ nanolayers can be involved in the catalytic action of oxidizing the entire carbon surface species to H_2_O and CO_2_ observed in our TDS spectra. At the same time, the relative [Ag]/[Sn] concentration is also subsequently decreased reaching a value of 0.15 ± 0.05. This is probably due to the subsequent Ag atoms' diffusion into the subsurface region of L-CVD SnO_2_ nanolayers. This is related to the fact, that the depth of Ag diffusion into the L-CVD SnO_2_ subsurface layer is larger than the XPS information depth (in average 3 mean free paths of approximately 4 nm).

**Figure 4:**
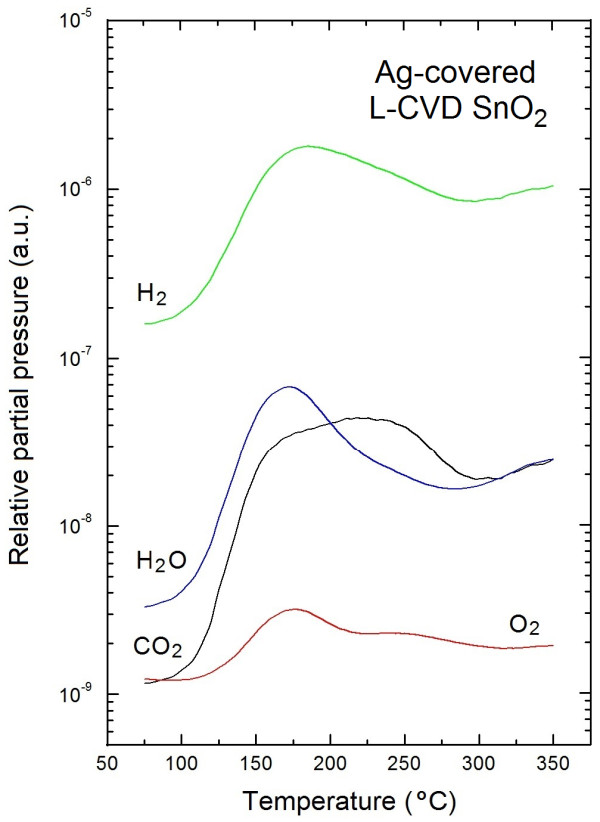
**TDS spectra of residual gases desorbed from Ag-covered L-CVD SnO**_**2 **_**nanolayers.**

All the obtained information on the evolution of surface chemistry of Ag-covered L-CVD SnO_2_ nanolayers are in a good correlation with the information obtained from TDS spectra shown in Figure 4.

The TDS spectrum in Figure 4 shows evidently that mostly molecular hydrogen (H_2_) was mainly desorbed from the Ag-covered L-CVD SnO_2_ nanolayers, with highest relative partial pressure at the level of almost 8 × 10^−7^ mbar at about 190°C. This experimental fact has not yet been described in the available literature to our knowledge. Probably, due to the porous morphology of these nanolayers observed with AFM [8], molecular hydrogen having the smallest dimension among all the residual gases easily penetrates the subsurface space of Ag-covered L-CVD SnO_2_ nanolayers and, for the same reason, can be easily removed during registration of TDS spectra. Of course, this observation looks as critical because H_2_ can affect the sensing mechanism at the surface of SnO_2_ gas sensors leading to a reduction of the SnO_2_. However, we did not observe this effect, probably for two reasons. Firstly, the relative molecular hydrogen partial pressure we observed during the registration of our TDS spectra is evidently smaller in comparison to the typical concentration in gas sensor experiments (parts per million level). Secondly, a reduction of the SnO_2_ by H_2_ can only be observed at evidently higher working temperature, as also observed in [12].

Moreover, from the TDS spectra shown in Figure 4, it is visible that apart from H_2_, the water vapor (H_2_O) and carbon dioxide (CO_2_) mainly desorbed from the air exposed Ag-covered L-CVD SnO_2_ nanolayers.

For H_2_O the highest relative partial pressure at the level of 7 × 10^−8^ mbar at about 180°C was observed and was one order of magnitude smaller than for the case of H_2_. In turn for CO_2_, there is a wider range of desorption temperature (150°C ÷ 240°C), and the highest relative partial pressure of about 6 × 10^−8^ mbar was observed at about 220°C. This probably means that C-containing surface contaminations are more strongly bounded to the internal surface of the air exposed Ag-covered L-CVD SnO_2_ nanolayers. This last observation was in a good correlation with an evident decrease (by factor of 3) of C contaminations from these nanolayers as determined by the subsequent XPS experiments (see Figures 1 and 3).

However, at this point it should be additionally explained that we have registered the TDS spectra only up to 350°C, because even higher temperature does not allow the complete removing of C from the surface of L-CVD SnO_2_ nanolayers. Instead, in such a condition the C exhibits a tendency to uncontrolled and undesired diffusion to L-CVD SnO_2_ nanolayers observed in our recent XPS depth profiling studies [6]. According to our observation, a common approach observed in literature is mistakenly neglecting a role of C contamination at the surface and inside the SnO_2_ thin films working as the gas sensors to different oxidizing gases. This is crucial, since these gases strongly affect the sensing mechanism at the surface of SnO_2_ gas sensors working in normal conditions. This is probably a reason that the highest sensitivity of SnO_2_ gas sensors is observed at about 200°C.

Finally, also the molecular oxygen (O_2_) desorbs from the air-exposed Ag-covered L-CVD SnO_2_ nanolayers during the registration of TDS spectra. However, at the evidently lowest partial pressure varying within one order of magnitude and reaching a maximum value of about 4 × 10^−9^ mbar at about 180°C. It means that the molecular oxygen (O_2_) is also rather weakly (physically) bounded at the internal surface of the air-exposed Ag-covered L-CVD SnO_2_ nanolayers. Moreover, it should be mentioned that probably part of molecular oxygen (O_2_) observed in TDS spectrum can appears also as a result of H_2_O dissociation at the surface of Ag-covered L-CVD SnO_2_ nanolayers, which was in a good correlation with the variation of [O]/[Sn] described above.

## Conclusions

Our comparative XPS, TDS, and AFM studies of Ag-covered L-CVD SnO_2_ nanolayers deposited on atomically clean Si(111) substrate and subsequently exposed to air showed the following:

As deposited L-CVD SnO_2_ nanolayers (20-nm thickness) covered with 1 ML of Ag consisted a mixture of tin oxide SnO and tin dioxide SnO_2_ with the relative [O]/[Sn] concentration of approximately 1.3.

After long-term dry air exposure of the Ag-covered L-CVD SnO_2_ nanolayers, they were still a mixture of tin oxide (SnO) and tin dioxide (SnO_2_) phases with slightly increased [O]/[Sn] ratio of approximately 1.55, related to the adsorption of oxygen containing residual air gases from the air; moreover, an evident increase of C contamination was observed with [C]/[Sn] ratio at approximately 3.5, whereas surface Ag atoms concentration was twice smaller.

After registration of TDS spectra, the non-stoichiometry of Ag-covered L-CVD SnO_2_ nanolayers goes back to 1.3, whereas C contamination evidently decreases (by factor of 3) but cannot be completely removed in this process. Simultaneously, Ag concentration subsequently decreased by factor of approximately 2, which was related to the diffusion of Ag atoms into the subsurface layers related to the grain-type surface/subsurface morphology, as confirmed by XPS ion depth profiling studies.

The variation of surface chemistry of Ag-covered L-CVD SnO_2_ nanolayers before and after registration of TDS spectra observed by XPS was in a good correlation with the desorption of residual gases like H_2_, H_2_O, O_2,_ and CO_2_ from these nanolayers observed in TDS experiments.

All the observed experimental facts testified the limited sensing application of L-CVD SnO_2_ nanolayers, corresponding to the long response/recovery times, for instance, in NO_2_ atmosphere, as was observed some years ago by group of Larciprete [13]. However, their electronic and sensing properties are still currently under investigation in our group.

## Abbreviations

AFM: atomic force microscopy; L-CVD: laser-enhanced chemical vapor deposition; ML: monolayer; NL: nanolayers; TDS: thermal desorption spectroscopy; UHV: ultrahigh vacuum; XPS: X-ray photoelectron spectroscopy.

## Competing interests

The authors declare that they have no competing interests.

## Authors' contributions

MK was involved in carrying out the XPS and TDS experiments, analyzing the experimental data and drafting the manuscript. LO conceived of the XPS and AFM study, and verified the manuscript. PK was involved in carrying out the TDS measurements. JS conceived of the study. All authors read and approved the final version of the manuscript.

## References

[B1] Göpel W Schierbaum K-D SnO_2_ sensor: current status and future progress Sensors Actuators 1995 9 1 12

[B2] Comini E, Faglia G, Sberveglieri G Electrical based gas sensors Solid State Gas Sensing 2009 New York: Springer 47 108

[B3] Carpenter MA Mathur S Kolmakov A Metal Oxide Nanomaterials for Chemical Sensors 2013 New York: Springer

[B4] Lantto V Mizsei J H_2_S monitoring as an air pollutant with silver-doped SnO_2_ thin-film sensors Sensors Actuators 1991 9 21 25

[B5] Kwoka M Ottaviano L Passacantando M Santucci S Czempik G Szuber J XPS study of the surface chemistry of L-CVD SnO_2_ thin films after oxidation Thin Solid Films 2005 9 36 42 10.1016/j.tsf.2005.04.014

[B6] Kwoka M Ottaviano L Passacantando M Santucci S Szuber J XPS depth profiling studies of L-CVD SnO_2_ thin films Appl Surf Sci 2006 9 7730 7733 10.1016/j.apsusc.2006.03.065

[B7] Kwoka M Waczynska N Koscielniak P Sitarz M Szuber J XPS and TDS comparative studies of L-CVD SnO_2_ ultra thin films Thin Solid Films 2011 9 913 917 10.1016/j.tsf.2011.04.185

[B8] Kwoka M Ottaviano L Szuber J AFM study of the surface morphology of L-CVD SnO_2_ thin films Thin Solid Films 2007 9 8328 8331 10.1016/j.tsf.2007.03.035

[B9] Wagner CD Riggs WM Davis LE Moulder JF Milenberger GE Handbook of X-ray Photoelectron Spectroscopy 1979 Eden Prairie: Perkin-Elmer

[B10] Maffeis TGG Owen GT Penny MW Starke TKH Clark SA Ferkela H Wilks SP Nano-crystalline SnO_2_ gas sensor response to O_2_ and CH_4_ at elevated temperature investigated by XPS Surf Sci 2002 9 29 34 10.1016/S0039-6028(02)02301-4

[B11] Kwoka M Ottaviano L Passacantando M Czempik G Santucci S Szuber J XPS study of surface chemistry of Ag-covered L-CVD SnO_2_ thin films Appl Surf Sci 2008 9 8089 8092 10.1016/j.apsusc.2008.03.019

[B12] Kwoka M Szuber J Czempik G X-ray photoemission spectroscopy study of the surface chemistry of laser-assisted chemical deposition SnO_2_ thin films after exposure to hydrogen Acta Physica Slovaka 2005 9 391 399

[B13] Larciprete R Borsella E De Padova P Perfetti P Faglia G Sberveglieri G Organotin films deposited by laser-induced CVD as active layers in chemical gas sensors Thin Solid Films 1998 9 291 295 10.1016/S0040-6090(97)01201-7

